# Bull Fertility and Its Relation with Density Gradient Selected Sperm

**DOI:** 10.22074/ijfs.2016.4721

**Published:** 2016-11-11

**Authors:** Lynda Allouche, Toufik Madani, Mohamed Mechmeche, Laetitia Clement, Allaoua Bouchemal

**Affiliations:** 1 Department of Biology and Animal Physiology, Faculty of Nature and Life Sciences, Ferhat Abbas Setif 1 University, Setif, Algeria; 2 Department of Agronomy, Faculty of Nature and Life Sciences, Ferhat Abbas Setif 1 University, Setif, Algeria; 3 National Center for Artificial Insemination and Genetic Improvement, Algiers, Algeria; 4 National Laboratory for Health Control in Breeding stock (LNCR), Allice, Maison Alfort, France

**Keywords:** Frozen Semen, Fertility, Bull

## Abstract

**Background:**

Sperm selection method is usually used to collect these cells for *in vitro*-assisted reproduction. Few studies reported the relationship of *in vivo* fertility and semen parameters after sperm selection; hence, the present study attempted to assess different semen parameters after post-thaw or sperm selection, using density gradient separation BoviPure®, to predict *in vivo* fertility.

**Materials and Methods:**

In this experimental study, frozen semen quality of four Montbeliarde bulls were assessed after post-thaw (PT) or after sperm selection (SSp), using density gradient separation BoviPure®, to predict the fertility rate in vivo. In addition to PT or SSp, semen was examined for concentration, motility, morphology abnormalities, viability, acrosome and plasma membrane integrities. Fertility was measured as non-return rates within 56 days after the first insemination (NRR) or as corrected NRR, expressed as CNRR, to the factors influencing fertility using linear mixed model. Non-parametric Kruskal-Wallis test was performed to compare semen parameter variables. Fertility rates were compared using Chi-square test. Pearson correlation analysis was used to test the relationship between CNRR and semen parameters. Data was analysed using SPSS package program, version 21.0.

**Results:**

Most of the examined bulls exhibited a high fertility rate (3/4 bulls, 62.1- 81.8% for NRR or 67.2-98.5% for CNRR). Fertility rate, expressed as CNRR, was significantly related to semen parameters after SSp, but not after PT. Thus, CNRR was increased with decrease of total motility, progressive spermatozoa and abaxial implantation frequencies after SSp (r=-0.999, P=0.001; r=-0.990, P=0.010; r=-0.988, P= 0.012, respectively); while, CNRR was decreased with decrease of SSp immotile spermatozoa (r=+0.995, P=0.005), underlying that maximal limit of determined immotile spermatozoa is 47%.

**Conclusion:**

High frequencies of total and progressive motility spermatozoa, and abaxial implantation in gradient selected sperm appear to be not favorable for fertility *in vivo*.

## Introduction

Artificial insemination (AI) is the cheapest and most applicable method of reproductive biotechnology around the world to select superior genetic of sires and dams (1). In semen production centers, quality control should be considered before selling it to livestock producers (2). Considering that usage of AI allows semen from one bull to be used for insemination into thousands of females, bull effects are paramount on herd genetics, dynamics, and production. Use of sperm from a low fertility (or infertile) bull leads to lower pregnancy rates, which then results in greater economic costs of housing these bulls and non-pregnant cows (3). Even though semen assay *in vitro* would be of great benefits in AI programs for determining bull fertility, it is unlikely feasible to evaluate a single sperm characteristic reflecting the real sperm fertilization capacity of the semen sample. 

Until now, no single laboratory test was able to accurately predict *in vivo* fertility; hence, potential bull fertility can be estimated from laboratory semen assessment with higher accuracy when a combination of several sperm analyses are performed *in vitro* (4). However, spermatozoa require capacitation before fertilization; mammalian spermatozoa must undergo epididymal maturation, capacitation and the acrosome reaction to fertilize an oocyte. Capacitation is possible even *in vitro* in the absence of reproductive-tract fluids and several compounds are known to induce capacitation *in vitro*. During capacitation, several biochemical modifications occur on the sperm surface; such changes are essential in permitting sperm-oocyte binding and the acrosome reaction (5). 

In the mid-1980s, it was not always clear, how specific sperm procedures impacted sperm to enhance *in vitro* fertilization (IVF) in the bovine. Effect could have been on capacitation, acrosome reaction, or both. Compositions of different media are used in oocyte handling, sperm preparation and IVF (6). Sperm selection is a term with many interpretations; however, it is generally used to describe methods for separation of spermatozoa for *in vitro*-assisted reproduction (1). The techniques of “swim-up” and “swim-down” were often used for sperm selection after washing by extension and centrifugation, filtration/gradient separation, or self-motility (7). 

Many scientists investigated the relationship between post-thaw sperm parameters and fertility (8-15); but, few studies reported results on the relationship between fertility *in vivo* and semen parameters after selection of fertile spermatozoa (15, 16). Thus, our research attempted to assess different semen parameters after post-thaw (PT) or after sperm selection (SSp), using density gradient separation BoviPure®, to predict fertility *in vivo*. 

## Materials and Methods

### Chemicals

All of the used chemicals were reagent grade and purchased from Sigma-Aldrich (St. Louis, MO, USA).

### Semen source and examination

In this experimental study, frozen semen quality of four Montbeliarde bulls (1-4) was examined. The examined straws were provided from the same batch which was used for AI and purchased from National Center for AI and Genetic Improvement (Algiers, Algeria). 

For each bull, four straws from one freezing batch, two straws after PT and two straws after SSp, were assessed for concentration, motility, morphology abnormalities, viability, acrosome and plasma membrane integrities. 

Semen straws were thawed for AI analysis at 37°C for 30 seconds, to assess different sperm parameters. As few spermatozoa are available after SSp, some manipulations were adapted to have enough spermatozoa for this observation. All sperm parameters were performed by the same operator.

### Density gradient selected sperm

Sperm selection was performed using a commercial product BoviPure® (Nidacon Laboratories AB, Göothenborg, Sweden) according to manufacturer's instruction. In sterile graduated centrifuge tube of 10 ml, 2 ml of BoviPure 80% Layer was placed. Next, 2 ml of BoviPure 40% was carefully added and incubated at 37°C. After thawing semen, straw was cut in one side and fixed in syringe, followed by cutting the second side. Semen was placed gently on the prepared gradient of BoviPure®. After centrifugation for 15 minutes at 300x g, supernatant was carefully removed up to 0.3 ml, and remaining semen suspension was subsequently mixed and evaluated.

### Motility evaluation

Thawed semen was diluted 1:4 in pre-warmed phosphate buffer saline (PBS, NaCl 0.138 M, KCl 0.0027 M, pH=7.4) containing freshly prepared 1% bovin serum albumin (BSA) in our laboratory. Spermatozoa were incubated at 37°C for 3 minutes, before motility assessment, in the laboratory of semen production of Wallonne Breeding Association (AWE, Belgium).

For assessment of individual motility, one drop was placed between slide and coverslip and observed on Sumsung monitor PC via a 295 camera Leica connected to a trinocular phasecontrast Leica DM 1000 microscope (Germany), equipped with a Leica warm stage (37°C). Spermatozoa were examined at magnification of ×400 after PT or ×200 after SSp. A total of 200 spermatozoa were observed in at least 10 different fields, each spermatozoa was categorised in one of the following three motility classes: progressive, non-progressive or immotile spermatozoa. The proportion of each motility class was then calculated regarding the total number of spermatozoa. Total motility frequency is the sum of progressive motility and non-progressive motility frequencies. 

### Sperm concentration

An aliquot of thawed semen was diluted 1:20 in 1% formaldehyde solution. Spermatozoa were counted in duplicate using a hemocytometer.

### Examination of spermatozoa viability and morphology 

Viability and morphology of spermatozoa were assessed by mean of eosin-nigrosin staining. The stain was prepared using 3.3 g of eosin Y, 20 g nigrosin, 1.5 g sodium citrate and they were dissolved in 300 ml of warmed distilled water adjusting to pH=6.8-7. The stain was then filtered and preserved at 4°C (17, 18).

Two drops (40 µl) of thawed semen were mixed with one drop (20 µl) eosin-nigrosin on the pre-warmed slide and incubated for 2 minutes at 37°C. A thin smear was made and air dried. At least 200 spermatozoa were observed under bright field and oil immersion (magnification: ×1000) using Leica DM 1000 phase contrast microscopy.

Abnormal spermatozoa were classified, according to the guideline of the previous report (19): primary abnormalities (proximal cytoplasmic droplets, pyriform heads, strongly folded or coiled tails, midpiece defects, maldeveloped, and craters), and secondary abnormalities (distal droplets, tailless heads, simple bent or terminally coiled tails, small or giant heads, abaxial implantations, and abnormal acrosomes) (17, 20). However, frequency of abnormal acrosome was assessed in other smear, according to description in the following procedure.

### Hypo-osmotic swelling test and acrosomal evaluation

Frequencies of normal acrosome and positive hypo-osmotic swelling (HOS) test for spermatozoa were determined as previously reported (21). In addition, plasma membrane integrity of spermatozoa was assessed using HOS test (22). HOS solution was prepared by dissolving 0.735 g sodium citrate and 1.351 g fructose in 100 ml distilled H_2_O (23). 

For assessment of positive spermatozoa HOS test after PT or SSp, 30 µl of semen sample was mixed to 300 µl pre-warmed HOS solution and incubated at 37°C for 45 minutes. After incubation, a drop of semen sample was placed in clean slide, covered with cover slip and examined under phase-contrast microscope. Positive spermatozoa for HOS were considered as coiled tail, due to intact plasma membrane. A total 200 spermatozoa were counted in different fields and percentage of positive spermatozoa for HOS test was then determined. 

However, assessment of normal acrosome was performed by fixing 50 μl of semen sample in 500 μl (PT) or 250 μl (SSp) of 1% formal citrate containing 2.9% (w/v) trisodium citrate dehydrate before capacitation *in vitro* (24). Thick smear was performed and at least 200 spermatozoa were observed at ×1000 magnification under oil immersion using a Leica DM 1000 phase-contrast microscopy to determine the frequency of normal apical ridge spermatozoa.

### Data collection and fertility measures

In this research, animal care protocol and all used procedures were approved by Algerian animal welfare laws and policies (law 88-08 of 1998, article 58).

In Algeria, dairy cattle are mostly present as small herds. Data were collected from dairy farms situated in Setif region, North-Eastern part of Algeria, involving 110 inseminations. Montbeliarde cows were inseminated after oestrus observation and estrus-insemination interval (EI) was then recorded for each cow. All cows included in this study were inseminated between 14.11 and 15.25 hours after estrus observation according to the routine insemination (12-24 hours) from estrus onset to avoid altering fertility. Inseminations were realized in both season (summer and fall), where maximum temperatures ranged between 44 and 45°C in summer and from 23 to 36°C in fall. Fertility was measured as non-return rates within 56 days after the first insemination (NRR) or as corrected NRR (CNRR) when NRR was statistically corrected for the factors influencing fertility.

### Statistical analysis

Semen variables are presented as means ± standard error and fertility as frequencies. Homogeneity of variance was examined by Levene’s test. As variances were unequal, nonparametric Kruskal-Wallis test was performed to compare semen parameter variables. Fertility was analysed as binary trait (yes or no) and compared using Chi-square test. NRR was collected and linear mixed model was conducted to correct NRR, expressed as CNRR, to the following factors: cow age (<3, 3-4 and >4 years), parity (1, 2 and ≥3), inseminator (1, 2), season (summer, fall) and proven AI service (4 bulls). Pearson correlation analysis was performed to test the relationship between CNRR and semen parameters; data normality was checked with Kolmogorov-Smirnov test. 

Differences were considered significant when P<0.05 and trends were discussed when P<0.10. All statistical analyses were performed using SPSS package program, version 21.0.

## Results

### Sperm motility and concentration 

Table 1 shows no significant difference between bulls for PT sperm concentration values and different motility frequencies. For SSp, semen concentration remains similar between bulls; the lowest total and progressive motility frequencies were observed in the bull 1 compared to the others, albeit these evident decreases are not statistically significant. 

**Table 1:** Semen concentration and motility (means ± SE) after post-thaw and selected sperm


				Motility frequencies (%)		
	Bulls (n)	Concentration(×10^6^)/Straw	Total motility	Progressive	Non progressive	Immotile

Post-thaw	1 (2)	25.71 ± 2.29	38.69 ± 5.46	29.57 ± 3.63	9.12 ± 1.83	61.31 ± 5.45
	2 (2)	24.13 ± 2.63	43.10 ± 6.20	31.10 ± 4.47	12.00 ± 1.73	56.90 ± 6.20
	3 (2)	22.37 ± 0.32	39.38 ± 1.55	30.51 ± 1.59	8.87 ± 0.04	60.62 ± 1.55
	4 (2)	30.79 ± 1.21	45.42 ± 1.37	35.82 ± 1.66	9.60 ± 0.28	54.49 ± 1.28
P values		0.160	0.682	0.367	0.321	0.475
Selected sperm	1 (2)	8.44 ± 1.35	53.34 ± 1.49	40.57 ± 1.03	12.77 ± 0.46	46.66 ± 1.49
	2 (2)	9.88 ± 0.74	77.57 ± 0.29	63.36 ± 2.14	14.24 ± 1.85	22.40 ± 0.28
	3 (2)	6.68 ± 0.39	72.85 ± 2.07	58.93 ± 2.27	13.92 ± 0.20	27.15 ± 2.07
	4 (2)	5.86 ± 0.15	62.73 ± 1.67	52.68 ± 3.26	10.05 ± 1.600	37.53 ± 1.41
P values		0.104	0.083	0.139	0.160	0.083


n; Number of straws from one freezing batch.

### Morphology abnormalities, hypo-osmotic swelling test positive and viable spermatozoa

There was no significant difference between bulls after neither PT nor SSp in the different morphology abnormality classes, HOS positive test and viable spermatozoa frequencies (Table 2).

### Fertility

Data of fertility (NRR or CNRR) are presented in Table 3. Field fertility of different bulls was varied widely, from low (51.9%) to high (62.1-81.8%) for NRR and from low (57.8%) to high (67.2-98.5%) for CNRR; so that fertility is the highest in the bull 1 and the lowest in the bull 2. Although NRR differences tend toward significance between different bulls (P=0.067), a significant difference in CNRR between bulls was observed (P=0.018).

Our results presented in Table 4 show that CNRR is negatively correlated to the following SSp parameters: total motility, progressive spermatozoa and abaxial implantation frequencies, respectively (r=-0.999, P=0.001; r=-0.990, P=0.010; r=-0.988, P= 0.012). A negative correlation trend was determined between frequency of acrosome abnormality and CNRR (r=-0.931, P=0.069). In contrary, CNRR were positively correlated to immotile spermatozoa frequency (r=+0.995, P=0.005).

**Table 2:** Percentage of morphology abnormalities. Hos-positive and viable spermatozoa (means ± SE) after post-thaw and selected sperm


	Post-thaw sperm (%)					Selected sperm (%)				
Bulls (n)	1(2)	2(2)	3(2)	4(2)	P	1(2)	2(2)	3(2)	4(2)	P

Proximal cytoplasmic droplets	0.00 ± 0.00	0.20 ± 0.20	0.00 ± 0.00	0.00 ± 0.00	0.392	0.00 ± 0.00	0.00 ± 0.00	0.00 ± 0.00	0.00 ± 0.00	1.000
Pyriformheads	0.23 ± 0.23	0.44 ± 0.44	0.39 ± 0.39	1.80 ± 0.99	0.344	2.51 ± 0.32	2.81 ± 0.73	1.25 ± 0.87	4.16 ± 2.61	0.608
Strongly folded/coiled tails	0.23 ± 0.23	0.44 ± 0.44	0.39 ± 0.39	1.80 ± 0.99	0.344	0.62 ± 0.26	0.92 ± 0.09	1.21 ± 0.07	2.17 ± 1.01	0.129
Midpiecedefects	0.45 ± 0.45	0.88 ± 0.88	0.97 ± 0.19	1.77 ± 1.46	0.809	0.00 ± 0.00	0.00 ± 0.00	0.00 ± 0.00	0.00 ± 0.00	1.000
Maldeveloped	0.00 ± 0.00	0.44 ± 0.44	0.00 ± 0.00	0.00 ± 0.00	0.392	0.00 ± 0.00	0.67 ± 0.16	0.00 ± 0.00	0.19 ± 0.19	0.116
Craters	0.22 ± 0.22	4.24 ± 1.45	0.00 ± 0.00	1.22 ± 0.72	0.161	0.00 ± 0.00	0.00 ± 0.00	0.00 ± 0.00	0.00 ± 0.00	1.000
Primaryabnormalities	1.13 ± 0.20	6.66 ± 0.56	2.18 ± 0.17	5.36 ± 0.52	0.083	3.13 ± 0.06	4.39 ± 0.66	2.47 ± 0.94	6.52 ± 3.44	0.367
Distal droplets	0.00 ± 0.00	0.00 ± 0.00	0.00 ± 0.00	0.00 ± 0.00	1.000	0.00 ± 0.00	0.00 ± 0.00	0.00 ± 0.00	0.00 ± 0.00	1.000
Tailless heads	1.14 ± 0.21	1.50 ± 0.27	1.77 ± 0.99	1.27 ± 0.34	0.908	3.89 ± 2.14	0.26 ± 0.26	1.41 ± 0.13	0.79 ± 0.02	0.083
Simple bent/terminal coiled tails	1.82 ± 0.89	1.26 ± 0.38	2.53 ± 0.96	1.52 ± 0.90	0.682	1.94 ± 0.19	2.9 ± 1.66	3.47 ± 0.8	1.55 ± 1.15	0.682
Small/giantheads	3.45 ± 0.75	6.22 ± 1.30	6.44 ± 0.24	4.40 ± 1.17	0.212	5.87 ± 0.17	7.47 ± 7.47	3.38 ± 0.83	11.54 ± 1.98	0.446
Abaxial implantations	0.24 ± 0.24	0.87 ± 0.46	1.95 ± 0.38	0.20 ± 0.20	0.148	0.18 ± 0.18	1.38 ± 0.14	1.26 ± 0.88	0.59 ± 0.19	0.198
Abnormal acrosomes	12.54 ± 2.58	10.32 ± 1.48	16.46 ± 1.68	13.15 ± 0.27	0.244	7.75 ± 1.83	26.32 ± 3.05	15.98 ± 0.57	14.81 ±2.31	0.112
Secondary abnormalities	19.17 ± 2.46	20.17 ± 0.18	29.13 ± 1.80	20.54 ± 0.00	0.193	25.89 ± 4.62	47.11 ± 7.17	30.41 ± 3.17	42.32 ± 1.26	0.139
Total abnormalities	20.30 ± 2.25	26.83 ± 0.73	31.31 ± 1.63	25.90 ± 0.52	0.104	29.02 ± 4.68	51.5 ± 6.51	32.87 ± 4.1	48.85 ± 4.7	0.129
Hos-positivespermatozoa	36.68 ± 5.68	43.41 ± 2.77	39.38 ± 10.2	52.08 ± 6.25	0.539	64.51 ± 1.88	72.97 ± 0.48	62.71 ± 1.17	61.40 ± 4.32	0.212
Viability	53.49 ± 1.22	40.77 ± 4.72	30.83 ± 8.76	35.86 ± 1.94	0.212	82.37 ± 4.47	73.58 ± 6.77	73.62 ± 6.53	67.62 ± 1.08	0.446


n; Number of straws from one freezing batch.

**Table 3:** Fertility of different bulls expressed as NRR or CNRR


Bulls	n	Fertility	
		NRR (%)	CNRR (%)

1	22	81.8	98.5
2	27	51.9	57.8
3	29	62.1	67.2
4	32	78.1	82.3
P values		0.067	0.018


n; Number of artificial insemination performed per bull, NRR; Non return rate- 56 days, and CNRR; Corrected non return rate-56 days.

**Table 4:** Relation between fertility (CNRR) and selected sperm parameters


Sperm parameters	CNRR	P values

Total motility	r= -0.999	0.001
Progressive motility	r= -0.990	0.010
Immotile	r= +0.995	0.005
Abaxial implantation	r= -0.988	0.012
Acrosome Abnormality	r= -0.931	0.069


r; Coefficient of Pearson correlation and CNRR; Corrected non return rate-56 days.

## Discussion

In the current study, cows were inseminated under difficult Algerian subtropical environment conditions, where the temperature was mostly high and cows received poor quality nutrition; these difficult rearing conditions could decline cow reproduction (25). The objective of current study is to research semen parameters, after post-thaw sperm or after density gradient selected sperm, as an indicator for fertility *in vivo* under farm management conditions.

In our study, no relationship was found between fertility and all semen parameters after PT, which is in agreement with the previous finding (8). Sperm motility is one of the parameters frequently evaluated in artificial insemination laboratories. There is no doubt that motility is an essential test for fertilization, regarding that spermatozoa should interact with oocyte for fertilization. None the less, motility of spermatozoa has been proven to be a poor indicator of fertility in frozen-thawed sperm, and poor relationships were found between fertility and post-thaw motility (9, 10). This finding is consistent with our results, while other authors reported a correlation between fertility and some post-thaw sperm parameters, such us sperm motility (11, 12), morphology, concentration and subjectively motility (13, 14), tail abnormalities and average path velocity (15).

Nevertheless, our results indicated that PT morphology abnormalities were <30% in most of bulls' thawed-semen (3/4), agreeing limited value considered by most of the artificial insemination centers. 

In cattle industry, field fertility is assessed by quantifying NRR. Selected bulls have usually an NRR, ranging between 60 and 80% (26). Most of the proven bulls tested in our study exhibited a high fertility (3/4 bulls, 62.1-81.8% for NRR or 67.2-98.5% for CNRR) which can explain lack of the relationship between fertility and PT semen parameters.

In our study, semen parameters after PT or SSp were not different between bulls. Interestingly, some semen parameters after SSp were related to the fertility, expressed as CNRR, but not to those after PT. Thus, CNRR was increased with decrease of SSp total motility, progressive spermatozoa and abaxial implantation frequencies. While CNRR was decreased, as SSp immotile spermatozoa was decreased, underlying that the maximal limit of assessed immotile spermatozoa is 47%. Gillan et al. (13), determined no correlation between fertility and subjectively or CASA- motilities after semen swim-up; while, a positive correlation was found between fertility and total motility-CASA after semen swim-up (15). We clearly demonstrated that spermatozoa after PT were different than those after SSp. After AI, spermatozoa need some modifications to be able to fertilize oocyte, such as capacitation. Indeed, Bovipure is used to clean sperm and select high quality of spermatozoa before fertilization *in vitro* in the bovine reproduction laboratories. There is persuasive evidence that capacitation of those spermatozoa participating in fertilization is actively and progressively coordinated within succeeding regions of the female tract and also coordinated with the time of ovulation. Under a normal sequence of biological events, mating would precede ovulation within particular time (27). Thus, sperm that reach an adequate capacitation state are released and able to move to the fertilization place (28, 29). 

Our study reveals that high frequency of total and progressive motility spermatozoa of SSp appears to be not favorable for fertility, suggesting that high motility shorten lifespan of the sperm; thus, when semen deposited in cervical uterus and undergo capacitation spermatozoa progress up rapidly in oviduct and reach the place of fertilization before ovulation. So that it cannot successfully fertilize the ovum. It seems that the male sperm, carrying Y chromosome, exhibit high motility, but has a shorter lifespan than the female sperm (sperm containing X chromosome) (30). However, Y sperm in the isthmus would achieve capacitation earlier than X sperm, releasing from the oviductal epithelium, and reach the fertilization place long before the ovulation, leading to death for most of these cells and fertilization could not occur in this case. However, delayed AI (≥30 hours) produces a significant deviation of the sex ratio towards the males (72.06%) (31). Further studies should be conducted to investigate relationship between motility and lifespan of sperm. Nevertheless, based on our results as well as those of the previous studies, and considering that cows were often inseminated between 12 and 24 hours, AI with high motility SSp should be delayed to improve fertility and produce male calves.

The results of the present study explain that the test of gradient selected sperm can mimic the conditions of female reproductive tract. Hence, evaluations of semen parameters after SSp reflect better semen quality *in vivo*. It was shown that selected spermatozoa in ram represented a different sperm sub-population, compared to the unselected one which could be related to the fertility *in vivo* (16).

Until now, no study reported relationship of fertility *in vivo* and semen parameters after BoviPure semen separation. However, it was demonstrated that *in vitro* cleavage rates and embryo production appeared to be superior, following BoviPure® compared to Percoll® separation (32, 33). Also, Data from studies performed *in vivo*, on humans, are scarce in comparison with those of studies in vitro (34).

Our study was carried out in small farms when cattle are bred under difficult condition; different works reviewed for this research are controversial and further studies merit to investigate the relationship between fertility *in vivo* and semen parameters after BoviPure® separation. 

## Conclusion

In the current research work, the highest fertility rate was observed in the bull with the lowest total motility, progressive spermatozoa and abaxial implantation as well as high immotile spermatozoa frequencies.

We highlighted that high percentage of progressive spermatozoa motility is an indicator for low fertility, so excess in sperm motility appeared to be unfavorable for fertility. Spermatozoa progress up rapidly in oviduct before the ovulation and they cannot successfully reach and fertilize an oocyte. Moreover, abaxial implantation frequency observed in SSp could be considered as sperm morphology abnormality, leading to fertility decline. 
